# Increased Case Notification through Active Case Finding of Tuberculosis among Household and Neighbourhood Contacts in Cambodia

**DOI:** 10.1371/journal.pone.0150405

**Published:** 2016-03-01

**Authors:** Fukushi Morishita, Mao Tan Eang, Nobuyuki Nishikiori, Rajendra-Prasad Yadav

**Affiliations:** 1 World Health Organization Regional Office for the Western Pacific, Manila, Philippines; 2 National Center for Tuberculosis and Leprosy Control, Ministry of Health, Phnom Penh, Cambodia; 3 World Health Organization Representative Office in Cambodia, Phnom Penh, Cambodia; Universidad Nacional de la Plata, ARGENTINA

## Abstract

**Background:**

Globally, there has been growing evidence that suggests the effectiveness of active case finding (ACF) for tuberculosis (TB) in high-risk populations. However, the evidence is still insufficient as to whether ACF increases case notification beyond what is reported in the routine passive case finding (PCF). In Cambodia, National TB Control Programme has conducted nationwide ACF with Xpert MTB/RIF that retrospectively targeted household and neighbourhood contacts alongside routine PCF. This study aims to investigate the impact of ACF on case notifications during and after the intervention period.

**Methods:**

Using a quasi-experimental cluster randomized design with intervention and control arms, we compared TB case notification during the one-year intervention period with historical baseline cases and trend-adjusted expected cases, and estimated additional cases notified during the intervention period (separately for Year 1 and Year 2 implementation). The proportion of change in case notification was compared between intervention and control districts for Year 1. The quarterly case notification data from all intervention districts were consolidated, aligning different implementation quarters, and separately analysed to explore the additionality. The effect of the intervention on the subsequent case notification during the post-intervention period was also assessed.

**Results:**

In Year 1, as compared to expected cases, 1467 cases of all forms (18.5%) and 330 bacteriologically-confirmed cases (9.6%) were additionally notified in intervention districts, whereas case notification in control districts decreased by 2.4% and 2.3%, respectively. In Year 2, 2737 cases of all forms (44.3%) and 793 bacteriologically-confirmed cases (38%) were additionally notified as compared to expected cases. The proportions of increase in case notifications from baseline cases and expected cases to intervention period cases were consistently higher in intervention group than in control group. The consolidated quarterly data showed sharp rises in all forms and bacteriologically-confirmed cases notified during the intervention quarter, with 64.6% and 68.4% increases (compared to baseline cases), and 46% and 52.9% increases (compared to expected cases), respectively. A cumulative reduction of case notification for five quarters after ACF reached more than -200% of additional cases.

**Conclusions:**

The Cambodia’s ACF with Xpert MTB/RIF that retrospectively targeted household and neighbourhood contacts resulted in the substantial increase in case notification during the intervention period and reduced subsequent case notification during the post-intervention period. The applicability of retrospective contact investigation in other high-burden settings should be explored.

## Introduction

Globally, tuberculosis (TB) continues to be a major public health problem. Despite the rapid and worldwide expansion of the DOTS Strategy to control TB for the past two decades, case notifications have stagnated since late-2000s, and 3 million incidence TB cases are estimated to remain undiagnosed or not notified each year [1]. While the routine TB services are essential particularly for case management, it has proven inadequate to control TB because available services are not always accessible to poor and vulnerable populations where TB often concentrates [2, 3]. In light of this challenge, a renewed interest in active case finding (ACF) as a complementary strategy to improve case detection has emerged [4–6]. However, evidence to suggest its benefits are yet fully mature [5].

Measuring direct yield would be the first step to assess the outcome of ACF [7]. Yet it does not illustrate the additional impact of ACF beyond the routine activity of the National Tuberculosis Program (NTP), besides it does not take case notification trend into consideration [8]. A recent rigorous systematic review exploring the benefits of systematic TB screening identified only three longitudinal studies and 14 cross-sectional studies that assessed contribution of screening to total case notifications [5]. The review presented moderate evidence to suggest that screening increases case notifications in the short term by showing a wide range of proportion of cases detected by the screening of all notified cases from 1% to 86% [5]. However targeting contacts did not contribute more than 9% of total cases [5]. Since the publication of this systematic review, several studies have documented the contribution of ACF to increased case detection [9–11]. Given the increasingly diversified approaches in ACF, however, different outcomes could be expected in different project contexts [12], highlighting the need of the individual project assessment to add to evidence-based policy guidance.

Moreover, there is little evidence on how ACF affects the subsequent case notification in intervention areas [5]. It could possibly lead to a reduction of case notification in the post-intervention period by detecting undiagnosed prevalent cases and averting secondary cases through interrupting further transmission. Although such sustained impact of ACF is epidemiologically expected and often hypothesized when assessing the medium-term population-level impact by using a mathematical model [13, 14], few studies have used the real project or country data to estimate the number of cases reduced after ACF implementation.

Cambodia is one of 22 countries with a high burden of TB, with the estimated prevalence rate of 715 per 100,000 population in 2013 [1]. The country has made considerable achievement in controlling TB since the re-launch of the NTP after a decade of constrained activities in the era of post-conflict [15]. Case notification, however, has stagnated since 2010. The number of all forms of TB notified in 2013 was 39,005 cases against the estimated incidence of 61,000 cases [1], showing approximately 22,000 cases (36%) were neither diagnosed nor notified [16].

To reach these missing cases, the Cambodia’s National Centre for Tuberculosis and Leprosy Control (CENAT), since 2005, has conducted nationwide active case finding (ACF) targeting household and neighbourhood contacts in poor communities alongside routine passive case finding (PCF). After 2012, the CENAT upgraded the ACF strategy by introducing Xpert MTB/RIF (Cepheid, Sunnyvale, CA, USA) to enable better diagnostic capacities especially for asymptomatic and sputum smear-negative patients. This approach has proven to be cost-effective [17] and yielded a substantial number of cases from a vulnerable age group with some indication of early case finding [18].

This study aims to investigate the impact of ACF on case notifications during and after the intervention period. Primary outcomes of interest include the estimated number and proportion of additional cases detected during the intervention period and cases reduced after the intervention.

## Methods

### Programmatic information

The Cambodia’s health system consisted of 77 operational districts (ODs) as of 2011. Each of these ODs served 100,000–200,000 populations. Of them, we selected 30 ODs that had high TB case notification rates for smear-positive TB (>125 per 100,000 population) and high poverty/access-barrier composite scores that were generated from seven indicators related to poverty, health access, or healthcare coverage in the Census and the Demographic Health Survey using a principal component analysis. These 30 ODs were randomly allocated into intervention and control groups. Each group had a population of approximately 2.9 million. For Year 1, ACF was implemented during February-December 2012 in 15 intervention ODs in parallel with the routine PCF, and the other 15 ODs continued with only routine PCF. For Year 2, ACF was implemented during May 2013-March 2014 in 15 ODs that had served as a control group in Year 1.

In catchment areas of the selected health centres in intervention districts, community volunteers and health workers conducted house-to-house visits of all smear-positive TB patients who had been registered for treatment during the preceding 2 years. In addition to household contacts, they also visited 10 nearest households from each index patient’s house in order to identify symptomatic neighbourhood contacts. During the visit, neighbourhood contacts were pre-screened for TB symptoms (cough, fever, weight loss, and/or night sweats of more than two weeks) on the spot by trained community volunteers and health workers. Neighbourhood contacts were included in the screening as they are likely exposed to infectious index cases within the same community that shared risk factors for TB. In places where the number of symptomatic suspects in the 10 nearest households was few, the pre-screening was extended to some of the next-nearest households within the same village. All of the household contacts regardless of TB symptoms and symptomatic neighbourhood contacts were invited to the prescheduled ACF session on a specific date in the nearest health centres. Two weeks prior to ACF session dates, CENAT-ACF team visited intervention sites to train existing healthcare staff and selected volunteers on how to gather and screen target population. On the day of ACF session, all participants were re-screened for TB symptoms at ACF sites by clinicians of the CENAT team, and underwent a chest X-ray (CXR). CXR films were developed immediately and evaluated by a radiologist of the team by classifying either normal or abnormal. Abnormal CXR findings were further classified as active TB, suspected TB, healed TB, or other abnormalities to facilitate clinical diagnosis. Individuals with abnormal CXR findings were asked to provide a sputum specimen for Xpert MTB/RIF testing. Those with MTB-positive results were reported as bacteriologically-confirmed TB. Diagnosis of bacteriologically-negative TB and extra-pulmonary TB was made by clinicians based on all available evidence on the same day of the ACF session. All detected patients initiated treatment immediately (within a few days in most cases) and they were managed by routine health services.

In contrast to ACF, TB cases registered under routine PCF were diagnosed according to national guidelines. Most cases were self-referral patients presenting to the health centres where a sputum smear microscopy is available. In case TB is still suspected in smear-negative subjects after anti-biotic trial, they should be referred to district health hospital for chest X-ray and subsequent assessment for diagnosis of smear-negative TB.

### Quantitative Data and Statistical Analysis

This study is a quasi-experimental cluster randomized design with intervention and control arms. We analysed TB case notification trends by using the routine TB surveillance data sourced from the NTP databases.

Multiple methods were used to identify additional TB cases. Firstly, historical baseline data were obtained from the previous year (Q1-Q4 2011 for Year 1, and Q2/2012-Q1/2013 for Year 2) and this was used to estimate the additional cases during the one-year intervention period. Secondly, trend-adjusted expected cases in the intervention period were estimated using the historical case notification data with reference to the past 8 quarters for Year 1 and 13 quarters for Year 2, and they were compared with the intervention period cases to compute the additional cases. The proportions of increase/decrease in case notifications from baseline cases and expected cases to intervention period cases were compared between intervention and control groups for Year 1. Thirdly, quarterly case notifications of all intervention districts were consolidated, separately for Year 1 and 2, through adjusting the intervention quarter in a way that different intervention quarters are aligned in the same quarter. From this quarter-adjusted notification data (for the past 28 quarters for Year 1 and 33 quarters for Year 2), trend-adjusted expected cases were calculated and historical baseline cases were also taken from the last quarter before the intervention quarter to estimate additional cases. This quarter-adjusted analysis enabled us to better extract the additionality of the intervention because the Cambodia’s ACF was a one-off event with its district-level implementation across different quarters within each implementation year, which dispersed the effect of the intervention in terms of time. All comparisons were made separately for all-forms and bacteriologically-confirmed TB.

In addition, using the consolidated data, we examined the trend in proportions of bacteriologically-confirmed cases among total notified cases to see any possible changes in clinical diagnosis during the intervention quarter.

Annualized case notification rates by quarter were also calculated to see the case notification trend controlling for changes in population. Population data were sourced from the Cambodian Health Management Information System at the Ministry of Health.

For Year1, we further assessed the effect of the intervention on the subsequent case notifications during 18 months after the intervention quarter to estimate the possible reduction of notified cases from expected cases. A cumulative reduction of case notifications and its proportion to the additional cases were calculated.

A linear regression model was applied to measure the trend-adjusted expected cases, and a local regression was used to see the trend in the proportion of bacteriologically-confirmed cases among total notified cases.

As this study used existing programme records and no personal identifying information was collected, ethical clearance was not required according to local regulations.

## Results

### Case notification before and during ACF

Table 1 summarizes the case notification data with the estimated additional cases and its proportion in intervention and control groups (No control group for Year 2 implementation). For the Year 1 implementation, 1467 cases of all forms (18.5%) and 330 bacteriologically-confirmed cases (9.6%) were additionally notified in comparison to trend-adjusted expected cases. During the same period, in contrast, all forms and bacteriologically-confirmed cases in the control districts decrease by 2.4% and 2.3%, respectively. The proportions of additional cases as compared to historical baseline cases were also consistently higher in intervention districts than in control districts (9.8% vs -13.2% for all forms and -1.3% vs -15.8% for bacteriologically-confirmed cases). In the intervention districts of the Year 2 implementation, case notifications of all forms and bacteriologically-confirmed cases increased by 23.4% and 9.7% [compared to baseline cases] and 44.3% and 38% [compared to expected cases], respectively.

**Table 1:** Summary of case notification and additional cases.

Group	Year	Actual cases				Trend-adjusted expected cases		Additional cases							
		Historical baseline cases		Intervention period cases				Compared to baseline cases (% change)				Compared to expected cases (% change)			
		All Forms	Bac+	All Forms	Bac+	All Forms	Bac+	All Forms		Bac+		All Forms		Bac+	
**Intervention districts**	**Year 1**	8551	3815	9389	3767	7922	3437	838	(9.8)	-48	(-1.3)	1467	(18.5)	330	(9.6)
**Control districts**	**Year 1**	8489	3207	7369	2701	7554	2766	-1120	(-13.2)	-506	(-15.8)	-185	(-2.4)	-65	(-2.3)
**Intervention districts**	**Year 2**	7222	2626	8910	2880	6173	2087	1688	(23.4)	254	(9.7)	2737	(44.3)	793	(38)

Bac+: bacteriologically-confirmed TB

**Fig 1:**
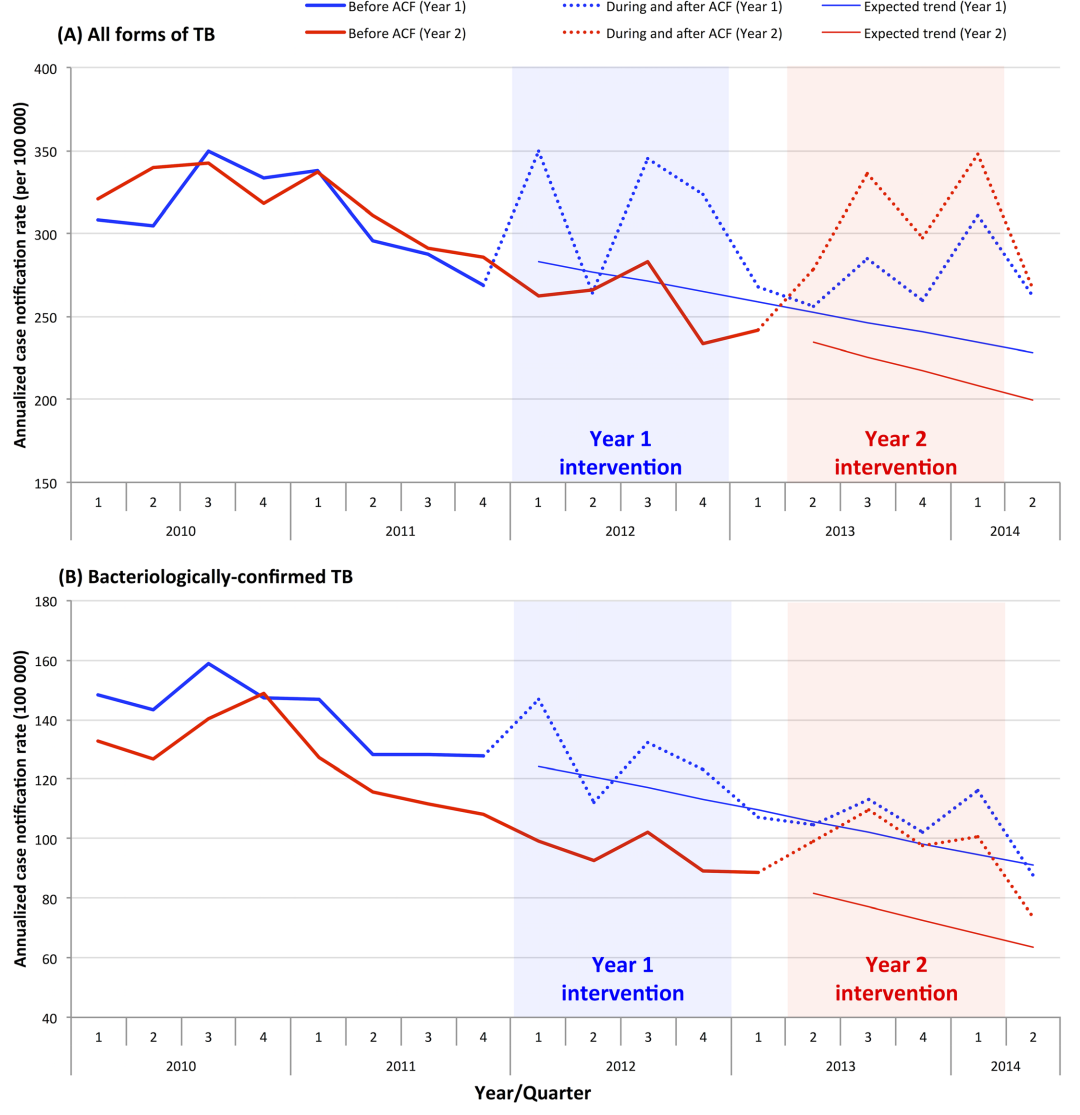
Annualized TB case notification rate by quarter.

The annualized case notification rates were compared between Year 1 and 2 districts and presented in Fig 1. Since 2010, there has been a decrease in historical case notification rates before intervention, generating a declining expected linear trend during and after intervention in both Year 1 and 2 districts. The similar trend was observed for all forms and bacteriologically-confirmed TB. During the intervention period, increased case notification rates were observed for both all forms and bacteriologically-confirmed cases but with a few fluctuations. During the Year 1 intervention period, a large increase of case notifications was not reported in the Year 2 districts which served as a control group during this period, suggesting case notifications had increased as a result of ACF.

### Consolidated case notification before and during ACF

**Fig 2:**
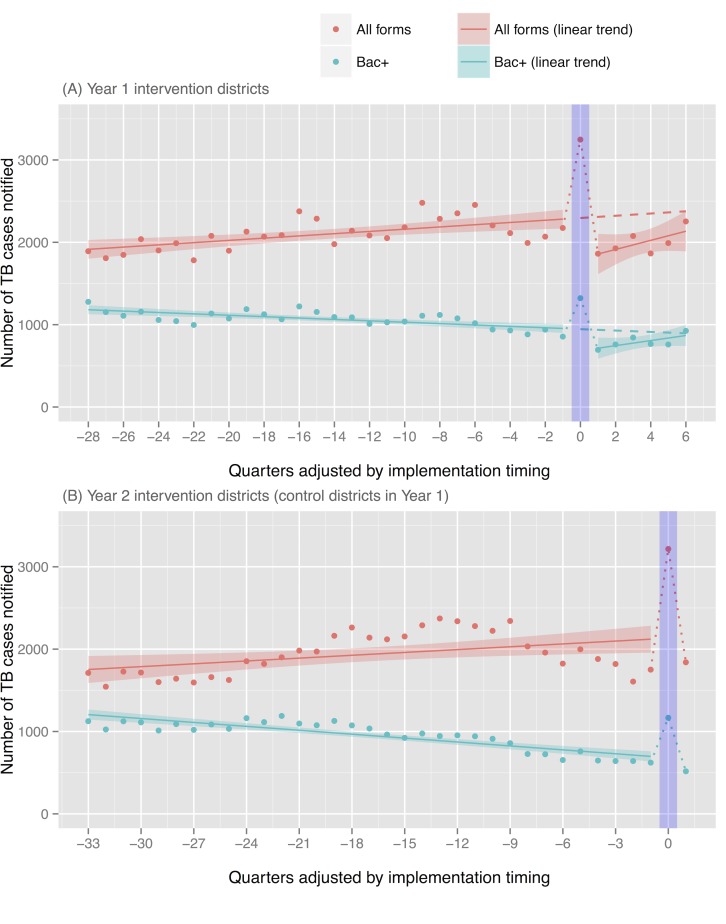
Consolidated case notification of all intervention districts, adjusted by implementation timing. Bac+: bacteriologically-confirmed TB.

Quarterly case notifications of all intervention districts were consolidated, separately for two different implementation years, through adjusting implementation timing in a way that the intervention quarter of different districts corresponds to 0 value of X-axis (Fig 2). (For example, Y value of -4 X-axis indicates a consolidated case notification reported in the quarter that is four quarters before the intervention quarter.)

For Year 1, case notification of all forms of TB ranged from 1738 to 2480 before the intervention quarter and it jumped up to 3247 in the intervention quarter (49.4% increase from the last quarter’s baseline) (Fig 2 and Table 2). Likewise, bacteriologically-confirmed cases sharply rose to 1322 after showing a slight declining trend before the intervention quarter (54.4% increase from the baseline). A substantial increase was observed as compared to trend-adjusted expected cases (41.5% for all forms and 39.7% for bacteriologically-confirmed cases). The same trend was observed in Year 2 with sharp rises in case notification during the implementation quarter (83.6% and 87.6% increases [compared to baseline cases], and 50.8% and 71.1% increases [compared to expected cases] for all forms and bacteriologically-confirmed cases, respectively). Summing up the Year 1 and 2 quarterly data, the proportions of increases were 64.6% and 68.4% [compared to baseline cases], and 46% and 52.9% [compared to expected cases] for all forms and bacteriologically-confirmed cases, respectively.

**Table 2:** Summary of quarter-adjusted case notification and additional cases.

Year	Actual cases				Trend-adjusted expected cases		Additional cases							
	Historical baseline cases from last quarter		Intervention quarter cases				Compared to baseline cases (% change)				Compared to expected cases (% change)			
	All Forms	Bac+	All Forms	Bac+	All Forms	Bac+	All Forms		Bac+		All Forms		Bac+	
**Year 1**	2174	856	3247	1322	2295	946	1073	(49.4)	466	(54.4)	952	(41.5)	376	(39.7)
**Year 2**	1752	622	3216	1167	2132	682	1464	(83.6)	545	(87.6)	1084	(50.8)	485	(71.1)
**Total**	3926	1478	6463	2489	4427	1628	2537	(64.6)	1011	(68.4)	2036	(46)	861	(52.9)

Bac+: bacteriologically-confirmed TB

**Fig 3:**
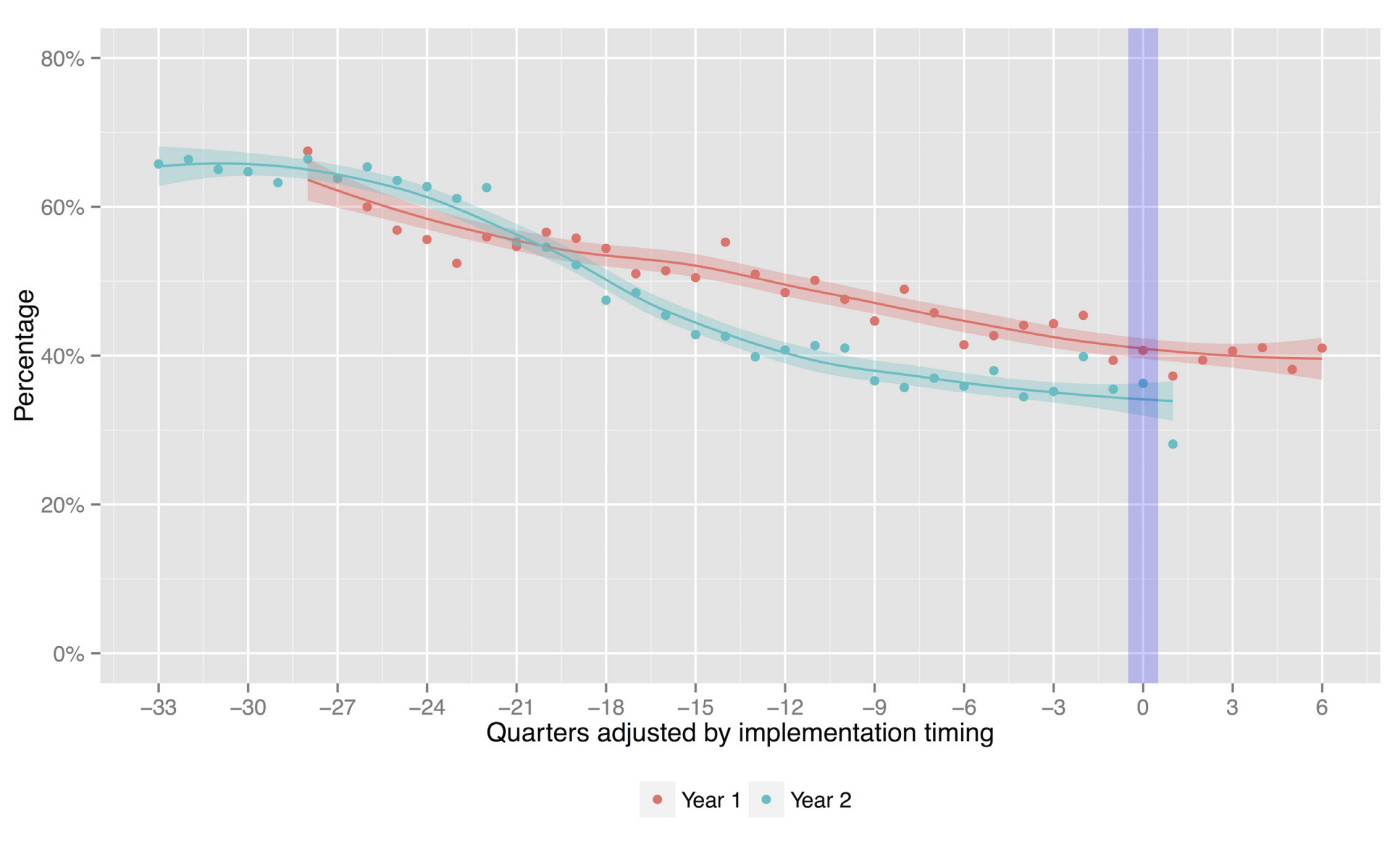
Proportion of bacteriologically-confirmed cases among total notified cases, adjusted by implementation timing.

The proportions of bacteriologically-confirmed cases among total notified cases in intervention districts are in a declining trend for both Year 1 and 2. They were 40.7% and 36.3% during the intervention quarter, respectively (Fig 3). Both rates fell within approximate 95% confidence bands for the local regression line.

### Consolidated case notification after ACF

Table 3 summarizes the case notification data by adjusted-quarter and the estimated additional/reduced cases during and after the intervention quarter for Year 1. Compared to trend-adjusted expected cases, 41.5% of all forms 39.7% of bacteriologically-confirmed cases were additionally notified during the intervention quarter. After the intervention quarter, case notifications of all forms had been lower than expected cases for the duration of six quarters. Similarly, case notifications of bacteriologically-confirmed cases had been lower than expected cases for the duration of five quarters after the intervention quarter although it slightly exceeded expected cases in the 6^th^ Quarter. Cumulative reductions of case notifications during 6 quarters (18 months) after the intervention were -2079 cases (-218% of additional cases) for all forms and -748 cases (-199% of additional cases) for bacteriologically-confirmed TB.

**Table 3:** Summary of case notification by adjusted-quarter during and after intervention quarter for Year 1.

Quarter adjusted	Trend-adjusted expected cases		Actual cases		Addition to/Reduction from expected cases (%)				Cumulative reduction after ACF (% of additional cases)			
	All Forms	Bac+	All Forms	Bac+	All Forms		Bac+		All Forms		Bac+	
0	2295	946	3247	1322	952	(41.5)	376	(39.7)	-	-	-	-
1	2309	937	1862	694	-447	(-19.3)	-243	(-26)	-447	(-47)	-243	(-65)
2	2322	929	1928	760	-394	(-17)	-169	(-18.2)	-841	(-88)	-412	(-109)
3	2336	920	2077	844	-259	(-11.1)	-76	(-8.3)	-1100	(-116)	-488	(-130)
4	2349	912	1865	766	-484	(-20.6)	-146	(-16)	-1584	(-166)	-634	(-169)
5	2363	904	1991	760	-372	(-15.7)	-144	(-15.9)	-1956	(-206)	-778	(-207)
6	2377	895	2254	925	-123	(-5.2)	30	(3.3)	-2079	(-218)	-748	(-199)

Bac+: bacteriologically-confirmed TB

## Discussion

Our analysis, with the use of multiple approaches in estimating additional cases, revealed that the Cambodia’s ACF increased case notification beyond what is reported in the routine PCF. In intervention districts, case notifications substantially increased during the intervention period as compared to historical baseline cases, trend-adjusted expected cases, and the control group. The proportions of increase in case notifications from baseline cases and expected cases to intervention period cases were consistently higher in intervention group than in control group. The analysis of quarter-adjusted consolidated notification data also showed exceptionally high increase in case notification during the intervention quarter. Furthermore, the cumulative reduction of case notification for five quarters after ACF reached more than -200% of additional cases notified during the intervention quarter.

Our results were consistent with several studies and reports that documented improved case detection through ACF [9–11, 19]. For instance, an intervention package employing community ACF initiatives in Ethiopia doubled case notification rate of smear-positive cases compared to the pre-intervention period and to the control areas [9]. Similarly, in India, a project of awareness drives and community-based ACF reported 11% increase in smear-positive notifications compared to pre-intervention period [11]. As the study setting, target population, screening method, and diagnostic tools and algorithms are different, they are not simply comparable each other. However, these results could demonstrate a large potential of ACF to improve case detection.

Furthermore, our results that showed 52.9% increase in bacteriologically-confirmed cases from trend-adjusted expected cases (in the consolidated data analysis) overrode the previous findings that targeting contacts did not contribute more than 9% of total case notifications [5]. Such successful outcome might be attributable to various factors including a large number of subjects screened by the intervention relative to routine PCF, underlying TB incidence among contacts in high-burden setting, as well as the tailored intervention approach with a combination of unique target selection and robust diagnostic tools and algorithms used in the Cambodia’s ACF.

The results from the National Prevalence Survey 2011 showed that the routine diagnostic algorithm (symptom screening and conventional sputum-smear examination) can detect only 14% of total prevalent cases in Cambodia, and other 84% can be detected through additional non-routine investigation by identification of asymptomatic TB suspects using CXR and/or bacteriological confirmation with culture [20]. Considering that Xpert MTB/RIF has a similar sensitivity as solid culture [21], such non-routine upgraded diagnostic tools and algorithms used in this intervention likely made substantial contributions to increased case detection. In fact, none of the contact investigation studies in the systematic review used non-routine enhanced diagnostic tools and algorithms [22–25].

Besides, retrospectively screening contacts of previous TB patients and inclusion of symptomatic neighbourhood contacts are also beyond what other programs commonly practice during routine contact investigation. This may thereby be important factors optimizing the effectiveness of the intervention. A study conducted in Hong Kong showed that the proportion of secondary cases with late development of active TB reported within 5 years was nearly two-fold higher than that of secondary cases detected by initial 3-months-period screening (1.24% vs 0.67%), demonstrating that considerable TB risk remains after initial contact investigation [26]. Similarly, in New York City, the substantial proportion of secondary cases were detected >9 months after the index case’s diagnosis during 4 years of follow-up [27]. In settings where initial contact screening is yet to be fully routinized, the proportion of secondary cases with late development of active TB could be much higher due to prolonged community transmission. This may be the case in most of high-burden countries with resource constraints including Cambodia [28] and further justify the effectiveness of retrospective contact investigation. A global policy on contact investigation prioritizes household contacts because non-household contacts are expected to be less influenced by the recent exposure to index cases and more influenced by general population risk factors [29]. A contact investigation study in Peru, indeed, showed a lower yield in neighbourhood contacts than in household contacts [30]. However, if neighbours are contacts, even though it is apparently casual, have socio-economic disadvantages and are symptomatic, they may carry sufficient risk factors and it could be reasonable to screen them together to make the best use of available opportunities and resources. Further investigation is required on possible differential yield between household and neighbourhood contacts.

Introduction of a rapid sensitive test could result in decrease in overall notifications in settings where many people are clinically diagnosed because clinicians are more convinced to rule out TB [7]. However, we observed increased notifications of both bacteriologically-confirmed and all forms cases during the intervention quarter without a notable change in the proportions of bacteriologically-confirmed cases among all forms. This implied that both bacteriologically-confirmed and all forms cases were nearly equally increased due to the intervention. Many of the symptomatic cases who would have been diagnosed as smear-negative TB without intervention could have shifted to bacteriologically-confirmed cases by the contribution of Xpert MTB/RIF. Likewise, many of the asymptomatic cases who would have been missed without intervention could have shifted to clinically-diagnosed cases by the contribution of CXR that has high sensitivity to detect early forms of TB [31]. Bacteriologically-negative clinically-diagnosed patients are likely early in their clinical course therefore diagnosing and treating them early also prevent further public health concerns.

Our results showing more than -200% cumulative reduction of case notification after ACF could indicate that the intervention detected not only prevalent cases earlier than they would have been detected without the intervention, but also cases that would have remained undiagnosed without the intervention. Technically, the latter can be defined as a true additional case [7]. To distinguish the two and approximate each of the cases is a challenge. However, if the intervention detected only cases that would have been captured by subsequent PCF, the cumulative reduction in subsequent case notifications should not be more than -100% of additional cases. Given that is not the case, the cases detected by the intervention include both true additional cases and early-detected cases.

Our short-term follow-up period does not explicitly allow us to see the impact of secondary cases averted since the impact of reduced transmission and incidence is anticipated several years after the intervention [7, 31]. However we assume that some of the secondary cases may be averted because the intervention substantially increased case notification of bacteriologically-confirmed infectious cases. Treatment initiation in those infectious cases should have, more or less, averted cases that would have resulted from rapid progression of recent infection without the intervention. It is difficult to estimate how many of those cases were averted without knowing TB incidence due to recent infection in this cohort. However, when the proportion of TB incidence due to recent infection is high, repeated ACF is expected to avert substantial number of cases [32]. For better understanding of it, context-specific TB transmission profiles need to be investigated.

This study has several limitations. Most importantly, observed case notifications could have been affected by other internal/external factors. First, the cases detected by the intervention could have been notified in neighbouring non-intervention districts, which might have influenced the case notifications. Such a spatial spill-over effect may be present but considered limited as all detected cases were actively followed-up by respective health facilities, and project records proved that cases lost before treatment were few. Second, a temporal spill-over effect, that the detected cases were notified in the following non-intervention quarter, most likely exists particularly in the districts with implementation towards the end of the quarter. This could have underestimated the number and proportions of additional cases and the subsequent reduction. Third, changes in local health system and phasing in and out of other TB case finding projects could have influenced case notifications both in intervention and control districts.

Although the national TB surveillance system has been progressively strengthened, the reliability of surveillance data is heavily dependent on local facility and staff capacity. The study results may not be generalizable in other settings outside Cambodia. However, this case finding strategy could be replicated in places with similar epidemiological and health service delivery profiles and resource availability.

In this study, the subsequent case notification gradually returned to pre-intervention level over the period of 5–6 quarters. This can be explained by the nature of the intervention that the Cambodia’s ACF was one-off events only in the selected health facilities. Hence, multiple rounds of screening may be needed to make a long-term sustainable impact at population level. To this end, the periodicity of the intervention should be carefully considered using the best available evidence.

## Conclusions

The Cambodia’s ACF with Xpert MTB/RIF that retrospectively targeted household and neighbourhood contacts resulted in the substantial increase in case notification during the intervention period and reduced subsequent case notification during the post-intervention period. Further investigation is required, with a longer follow-up period, on the periodicity of the intervention that has a sustained impact at community- and population-level. Furthermore, the applicability of retrospective contact investigation in other high-burden settings should be explored.

## Supporting Information

S1 Dataset Quarterly TB case notification data in selected operational districts in Cambodia: 2005–2014. (XLSX)
